# Use of a Plasma-Sprayed Titanium-Hydroxyapatite Femoral Stem in Hip Arthroplasty in Patients Older than 70 Years. Is Cementless Fixation a Reliable Option in the Elderly?

**DOI:** 10.3390/jcm10204735

**Published:** 2021-10-15

**Authors:** Nicola Piolanti, Elisabetta Neri, Enrico Bonicoli, Paolo Domenico Parchi, Stefano Marchetti, Mario Manca, Luca Bonini, Lorenzo Banci, Michelangelo Scaglione

**Affiliations:** 1 Ortopedia e Traumatologia I, Azienda Ospedaliera Universitaria Pisana, 56126 Pisa, Italy; elisabetta.neri@gmail.com (E.N.); enrico.bonicoli@libero.it (E.B.); parchip@tiscali.it (P.D.P.); marchettys@gmail.com (S.M.); michelangelo.scaglione@gmail.com (M.S.); 2 Ortopedia e Traumatologia, Ospedale della Versilia, 55049 Viareggio, Italy; mario.manca@uslnordovest.toscana.it (M.M.); bonluca@gmail.com (L.B.); 3 Permedica Orthopaedics, 23807 Merate, Italy; lorenzo.banci@permedica.it

**Keywords:** cementless stem, total hip arthroplasty, hip hemiarthroplasty, survival, plasma-spray, porous titanium, hydroxyapatite, elderly patient

## Abstract

Background: Although cementless implants are increasing in popularity, the use of cementless femoral stems for total hip arthroplasty (THA) and hip hemiarthroplasty (HH) in elderly patients remains controversial. The aim of this study was to report the outcomes of a cementless stem used in a large multicentric cohort of elderly patients receiving elective THA and HH for displaced femoral neck fracture. Methods: A total of 293 patients (301 hips) aged 70 years or older (mean age, 78 years; range, 70–93) who received the same cementless plasma-sprayed porous titanium–hydroxyapatite stem were retrospectively evaluated after primary THA and HH to investigate stem survival, complications, and clinical and radiographic results. Results: Cumulative stem survival was 98.5% (95% CI, 96.4–99.4%; 91 hips at risks) with revision due to any reason as the end-point at 10-year follow-up (mean 8.6 years, range 4–12). No stem was revised due to aseptic loosening. The mean Forgotten Joint Score was 98.7. Radiographically, the implants showed complete osseointegration, with slight stress-shieling signs in less than 10% of the hips. Conclusion: The use of cementless stems was proven to be a reliable and versatile option even in elderly patients for elective THA and HH for femoral neck fracture.

## 1. Introduction

Total hip arthroplasty (THA) is now considered an effective and reliable surgical procedure for the treatment of end-stage hip osteoarthritis [1]. It is well known that the number of people requiring THA is constantly growing and that the average life expectancy and patient expectations are rising; thus, the long-term survival and safety of prosthetic implants are becoming even more relevant requirements, particularly for elderly patients [2,3].

Cementless implants represent the standard in conventional primary THA for the general patient population [4,5], but in elective surgery, there is controversy regarding the use of cementless femoral stems rather than cemented stems in elderly patients because of poor-quality clinical evidence [4,6]. The best femoral component fixation method also remains unclear in hip hemiarthroplasty (HH) for acute femoral neck fracture [7,8].

Cementless stems are at higher risk of design-related complications, such as femoral fractures, thigh pain, and stress shielding. The use of cementless stems in primary THA reveals higher revision rates than those of cemented stems in patients older than 75 years with primary osteoarthritis, especially in women [9,10,11], and this trend is also reflected in HH [12]. The reason for the higher revision rate associated with cementless fixation can be mainly attributable to the increased risk of early periprosthetic fracture [13]. However, cementless stem fixation offers relevant advantages over cemented fixation, such as a reduced operative time, reduced cardiopulmonary perioperative complications, excellent long-term survival, bone sparing designs, and easier revision surgery in the case of early failure [4,7,14,15].

However, despite these premises, the use of cementless stems in elective THA is gaining increasing popularity worldwide in the clinical practice, especially for elderly patients, with promising results [3,4,9,11].

In the Tuscany region of Italy, since 2008, we have largely used a cementless stem with a double coating of plasma-sprayed titanium (Ti) and hydroxyapatite (HA) both in elective surgery and in trauma surgery for acute femoral neck fracture.

With these considerations, the question we aim to answer is as follows: is this cementless stem a viable option even when it is used in elderly patients in elective arthroplasty and in total or partial arthroplasty for displaced femoral neck fracture?

Thus, the purpose of this retrospective, multicenter, observational study was to report midterm outcomes of this plasma-sprayed Ti/HA stem used in a large consecutive series of patients of 70 years or older undergoing elective primary THA and THA or HH for displaced fracture of the femoral neck.

## 2. Materials and Methods

Three tertiary care public hospitals within the Tuscany region of Italy were selected as investigation sites for the study: Azienda Ospedaliera Universitaria Pisana, Ospedale della Versilia, and Ospedale di Livorno. Since 2008, in these three centers, a plasma-sprayed Ti/HA cementless femoral stem has largely been used in elective primary THA and HH for femoral neck fracture.

Inclusion criteria were patients who were 70 years old or older at the time of surgery; having a THA or HH with the same cementless plasma-sprayed femoral stem between 2008 and 2016 in these centers with, as indications, primary or secondary osteoarthritis, femoral head osteonecrosis, or subcapital displaced fracture of the femoral neck (Garden classification 3 or 4 [16]); and a minimum 4-year follow-up.

The primary study end-point was defined as the cumulative probability of survival of the femoral stem with revision for all reasons and for aseptic loosening. Secondary study end-points were major revisions and complications, and clinical and radiographic outcomes.

**Figure 1:**
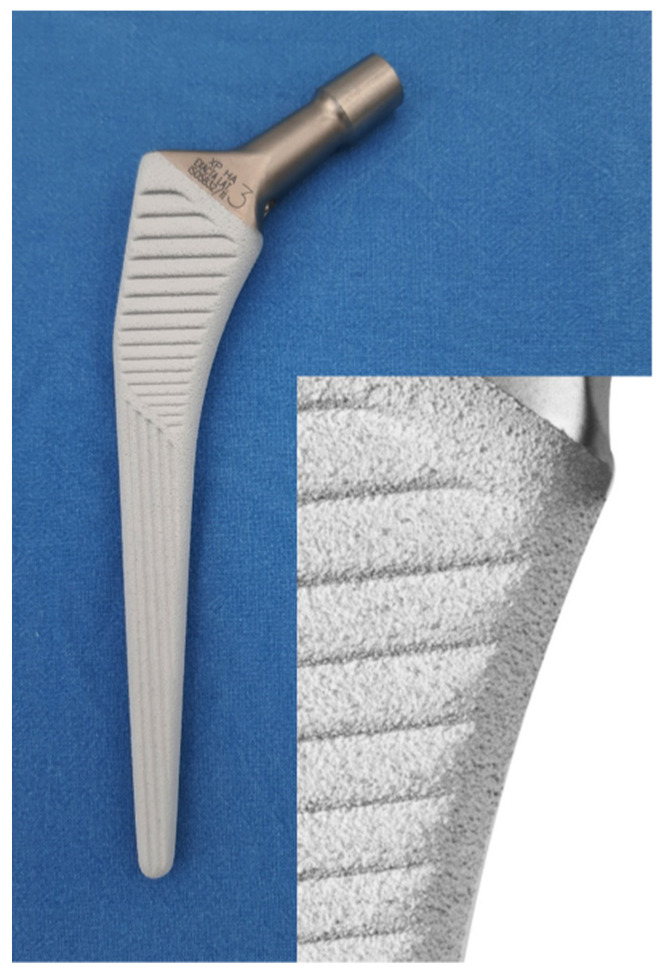
Image of the Exacta HAX-Pore^®^ femoral stem (lateralized version with +6 mm offset) and details of the plasma-sprayed porous titanium and hydroxyapatite double coating, developed by Permedica Orthopaedics, Merate, Italy.

The femoral implant used in all procedures was the Exacta HAX-Pore^®^ stem developed by Permedica Orthopaedics, Merate, Italy. The Exacta HAX-Pore^®^ stem is a cementless plasma-sprayed titanium stem with a straight, double-tapered design with a blunt rectangular cross-section and a rounded distal tip. The stem features a double-layer coating with open-pore 300 μm Ti and additional 50 μm HA, plasma-sprayed over a grit-blasted titanium alloy (Ti6AI7Nb), which provide an implant surface roughness of Ra ≥ 21 μm (Figure 1).

To increase primary stability and the bone-to-implant contact area, longitudinal grooves are present in the distal two-thirds of the stem and transversal grooves in the proximal third.

Surgical procedures were performed by five experienced senior surgeons (M.L., N.P., E.B., M.S., and M.M.) and their junior trainees. Each senior surgeon performs more than 100 hip arthroplasties per year, with an average of 20 Exacta stem implants per year. A posterior lateral surgical approach was used in all procedures with joint capsule repair when possible. The femoral canal was prepared with rasps that have bone-cutting teeth on the medial/lateral and anterior/posterior sides, allowing optimal fit between the stem and cortical bone.

This study was conducted by examining medical records, outpatient reports, and radiographic images of the patients undergoing surgery using the Exacta Hax-Pore^®^ stem. Surgery registers were evaluated to collect information on any intraoperative complications. Patients were contacted by telephone and then invited to a clinical and radiographic follow-up. Data according to the protocol were collected by N.P., E.B., P.D.P., S.M., L.B. (Luca Bonini), and E.N.

This study was approved on April 2019 by the Ethics Committee of Pisa with protocol number 14451. The study was conducted in accordance with the ethical standards laid down in the 1964 Declaration of Helsinki and its later amendments. All patients enrolled in the study gave their written informed consent.

### 2.1. Patient Selection

**Figure 2:**
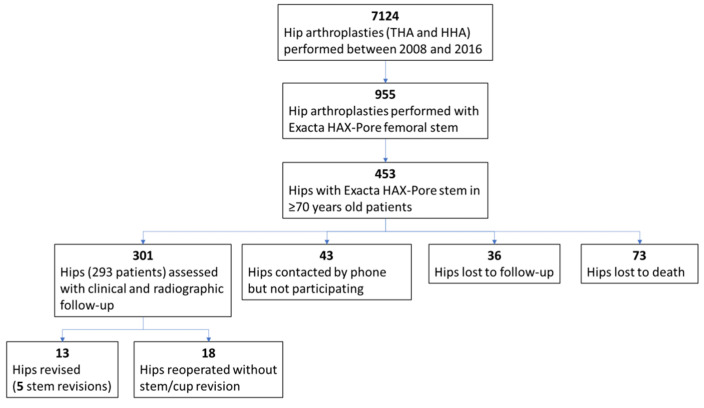
Study flow diagram.

Through a hospital database search, a total of 7124 THA and HH procedures were performed from 2008 to 2016, of which 955 were performed using the Exacta HAX-Pore^®^ stem. Out of these 955 implants, 453 were identified to be implanted in patients of 70 years of age or older. A total of 73 hips were lost because of death, 36 hips were lost to follow-up, and 43 patients were contacted about their hips by telephone but refused to participate, leaving 301 hips (293 patients) available for follow-up evaluation (Figure 2).

### 2.2. Clinical Evaluation

Clinically, patients were evaluated with the new Forgotten Joint Score (FJS) [17], a patient-reported outcome score administered at follow-up visit or by telephone interview to those patients unable to return to the hospital. Patients treated at other centers were also asked to provide information regarding the possible occurrence of complications.

### 2.3. Radiographic Analysis

Radiographic analysis was performed on the more recently available radiographs. If the available radiographs were obtained earlier than one year before the beginning of the study, new radiographs of the hip were produced at the study follow-up visit. The anteroposterior (AP) and lateral radiographs of the hips were independently evaluated by two authors who were not involved in the surgeries (L.B. (Luca Bonini) and P.D.P.). Any disagreement regarding radiographic evaluation was resolved by further evaluation of the radiographic parameter by one senior author (E.B.) to achieve consensus for all parameters.

The radiographs were examined for the following factors:

(1) Periprosthetic radiolucent lines [18] and osteolysis, defined as an area of localized progressive bone resorption or endosteal erosion [19], allocated according to the Gruen zones 1–14 [20].

(2) Bone hypertrophy, defined as a thickening of the periprosthetic bone [19] and allocated according to the Gruen zones 1–14.

(3) Stem subsidence ≥ 2 mm, considered as a significant risk factor of future implant loosening. This was evaluated by comparing the post-surgery AP radiograph and last follow-up AP radiograph [21]. In order to evaluate the subsidence of the stem using OrthoView, the distance between the tip of the greater trochanter and the line drawn between the trochanteric shoulder tip and the medial proximal groove near the neck was measured.

(4) Pedestal formation, defined as the shelf of endosteal new bone at the stem tip partially or completely bridging the intramedullary canal [22].

### 2.4. Statistical Analysis

Statistical analysis was performed using GraphPad Prism 8.0 statistical software (San Diego, CA, USA). Continuous variables were reported as mean ± standard deviation, and dichotomous variables were reported as numbers and percentages. The Mann–Whitney test was used to analyze differences between non-parametric variables. Implant cumulative probability of survival was determined according to the Kaplan–Meier method with a 95% confidence interval. Lost to follow-up cases were included as censored cases in survival analysis.

## 3. Results

The study cohort included 301 hips of 293 patients, on which 35 (11.6%) HHs were performed for displaced femoral neck fracture and 266 (88.4%) THAs (7 patients had undergone bilateral THA) were performed for primary or secondary osteoarthritis (227 hips, 75.5%), femoral head osteonecrosis (17 hips, 5.6%), and displaced femoral neck fracture (22 hips, 7.3%). The stem was implanted in femur Dorr type A in 24 hips (8%), type B in 124 hips (41%), and type C in 153 hips (51%). Regarding patient demographic data, 223 were female and 70 were male; the mean age at the time of surgery was 78 years, with a range from 70 to 93. Considering the design of the study, only patients with complete adhesion to the inclusion criteria were considered. Mean follow-up was 8.6 years (range, 4 to 12 years).

As an acetabular component, the Permedica Jump System HAX-pore^®^ press fit cup was used in 225 hips, the Permedica Jump System Cooper threaded cup in 24, the Stryker Trident System cup in 14, and the Lima Delta cup in 3. In all THA procedures, a 32 mm or 36 mm ceramic femoral head was used coupled with a ceramic or a vitamin E-blended, moderately cross-linked ultrahigh molecular weight polyethylene acetabular insert.

### 3.1. Complications

An intraoperative femoral fracture, i.e., calcar fissure, occurred during stem implantation in Dorr type B femurs in three (0.9%) female patients, two with osteoarthritis and one with femoral neck fracture: one femoral fracture was intraoperatively not recognized and afterwards was treated at 10 days with stem revision and cerclage of the femur, while the other two fractures were intraoperatively treated with femoral cerclage without any further complications.

**Figure 3:**
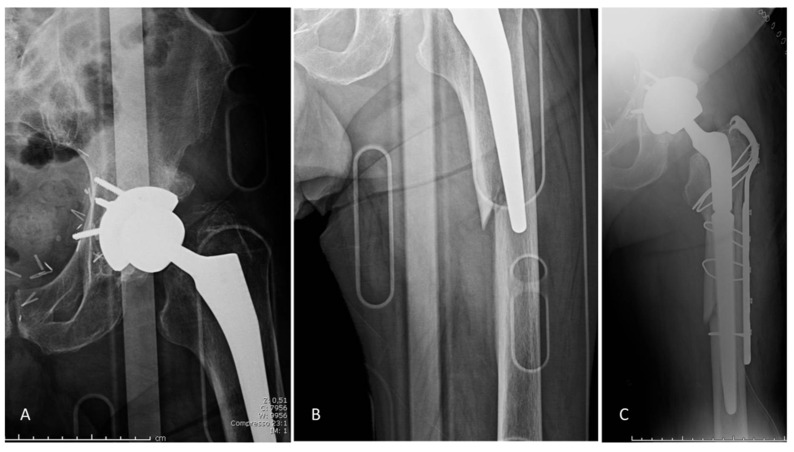
(**A**,**B**) Postoperative B2 periprosthetic fracture after trauma, which was successfully treated with stem revision with an uncemented, long modular revision stem and osteosynthesis by cerclage and fixation plate (**C**).

A total of 11 (3.6%) postoperative periprosthetic fractures occurred in 7 type C and 4 type B femurs, all due to traumatic events: 3 occurred after HH in osteoporotic patients with femoral neck fracture and 8 after THA in patients with osteoarthritis. Fractures were classified as type A in two hips, B1 in eight hips, and B2 in one hip according to Vancouver classification [23]. Nine type B1 fractures were treated by synthesis with plates, as the stem was found to be stable, and one type B2 fracture was treated by synthesis with plate and stem removal (Figure 3).

Periprosthetic infection was detected in 12 hips (3.9%): four hips with an early infection were successfully treated by debridement, antibiotics, and implant retention; three deep infections required two-stage revision; and the remaining five patients underwent chronic antibiotic therapy due to severe comorbidities compresence.

Prosthetic dislocation occurred in a total of nine hips (2.9%). Two dislocations occurred after HH and required conversion to THA, leaving the stem in situ. Hip reduction under narcosis was performed in two patients with THA without any other subsequent episodes, and the other five dislocations after THA required revision of the acetabular component only.

### 3.2. Implant Survival

Overall, 13 THAs were revised, of which five required stem revision and nine acetabular revision. An additional 18 hips were reoperated on without stem or acetabular cup removal.

**Figure 4:**
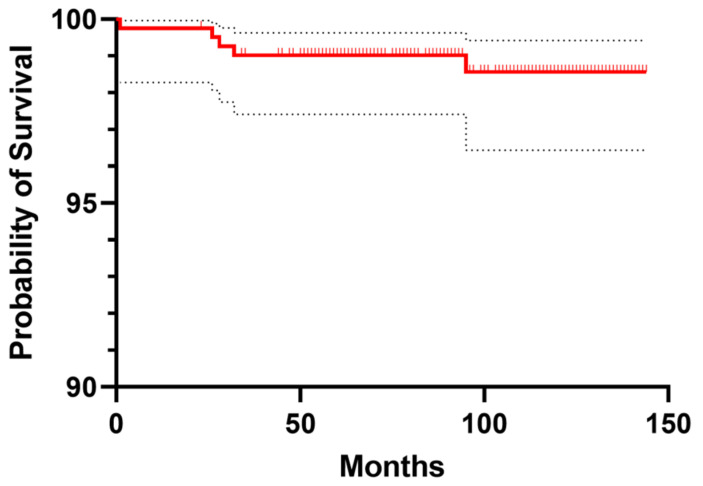
Kaplan—Meier cumulative survival of the femoral stem with femoral stem revision due to any reason as the end-point (red line). Dotted lines represent 95% confidence interval values.

The cumulative survival of the stem was 98.5% (95% CI, 96.4–99.4%) with stem revision due to any reason as the end-point at 10-year follow-up (91 hips at risk; Figure 4). Stem survival was 100% with stem revision due to aseptic loosening as the end-point.

### 3.3. Clinical Analysis

In patients without complications, the mean FJS was 98.7, with a range from 37 to 100. Five patients reported a negative FJS score (<70), stating a certain degree of discomfort regarding the prosthesis: in three of these patients, the index diagnosis was femoral neck fracture. Despite the poor clinical outcomes, the implant was radiographically stable in these patients.

### 3.4. Radiological Analysis

**Figure 5:**
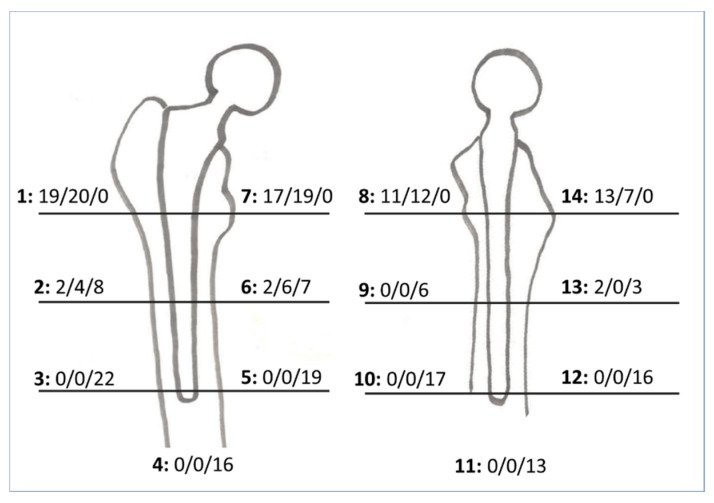
Radiographic assessment of the femoral stem according to Gruen zones 1 to 7 on anterior/posterior radiographs for 301 hips with complete available radiographic follow-up and zones 8 to 14 on lateral radiographs for 154 hips. In the figure, the numbers from left to right refer, respectively to: Gruen zone, number of hips with presence of radiolucency lines, number of hips with periprosthetic osteolysis, number of hips with cortical hypertrophy.

Radiographic evaluation was performed on AP radiographs for all 301 hips, with the lateral view available for 154 hips. Overall, there was a substantial degree of agreement among radiographic parameter assessments. In Figure 5, radiographic analysis results for radiolucent lines, osteolysis, bone hypertrophy, and formation are summarized.

**Figure 6:**
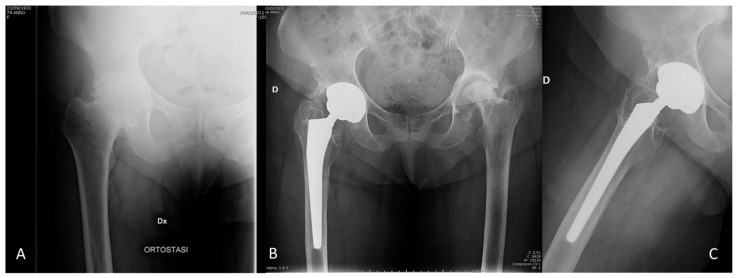
Ten-year radiographic follow-up of a THA performed in a woman aged 79 years with hip osteoarthritis, showing excellent osseointegration around the stem, lack of radiolucent lines, and minimal signs of stress-shielding with slight distal cortical hypertrophy and pedestal formation. (**A**) Preoperative AP radiograph of the affected hip. (**B**,**C**) AP and lateral radiographs at 10-year follow-up. A Brooker 3 heterotopic ossification was detectable laterally inside the capsule. D means right side.

All the identified radiolucency lines were evaluated as slightly ≤1 mm. Thin radiolucent lines and linear osteolysis were found in 19 hips (6%) in proximal zones. Cortical hypertrophy was identified in 22 hips (7%) in distal zones. Incomplete and complete bone pedestal was found below the stem tip in 16 hips (5%). The stem showed excellent osseointegration with direct contact with the cortical and cancellous bone in more than 90% of hips (Figure 6).

Stem distal migration ≥ 1 mm was detected on radiographic follow-up in 11 hips (3.6%), of which 5 (1.6%) showed subsidence ≥ 2 mm. These 11 hips with a mean stem subsidence of 1.6 mm (range: 1.0–2.4 mm) remained completely asymptomatic and included 4 Dorr B femurs and 7 Dorr C femurs, of which 2 occurred after periprosthetic fracture.

## 4. Discussion

There has been a worldwide trend toward cementless fixation in THA over the last two decades, and even countries where the traditional use of cement is well established have not been excluded from this trend [9,14].

Facing the increasing average life expectancy of the population, the growing patient expectations following THA, and the higher quality of healthcare and medical device standards, the long-term survival of hip prostheses is becoming even more essential [2].

In primary THA, implant loosening represents the most common reason for failure after midterm follow-up, showing a linear increase per follow-up year [3,10].

Revision for aseptic loosening is low in cementless THA, and, in particular, revision of cementless stems is the lowest in younger patients, who would be expected to have higher physical demands with higher failure rates secondary to loosening [14,15].

One of the most commonly used cementless stem designs is the Corail stem. While some registry-based studies have suggested that HA appears to have no significant clinical advantage in stem loosening [4], the literature is consistent regarding the excellent long-term survivals of the Corail stem [24,25,26,27]. Composite coatings of plasma-sprayed Ti particles with additional over-sprayed HA were developed to provide stronger coating adhesion strength to implant substrates and increased implant surface roughness [28]. Moreover, the first porous Ti coating, together with the external layer of HA, showed a synergic combination promoting fast implant osseointegration and stronger mechanical fixation with bone [29]. Some recent studies have reported excellent mid- to long-term stem survivals with the Polarstem, a Corail-design plasma-sprayed stem with 180 μm porous Ti and 50 μm HA, suggesting that this type of double Ti/HA plasma-sprayed coating could massively reduce the risk of implant aseptic loosening [30,31,32].

A similar trend towards cementless femoral fixation in THA has also occurred in aged patients [4,9]. Some studies in elderly patients have been conducted with short- and midterm follow-up on different cementless stems, with consistently satisfactory outcomes, but there is still poor clinical evidence [4]. Zimmerer et al. and Ahmad et al. recently reported 98% and 97.4% survival rates at 6-year follow-up for Corail and Polarstem, respectively, after THA in patients over 75 years [33,34].

The present study reported an excellent survival rate of the plasma-sprayed porous Ti/HA Exacta HAX-Pore^®^ femoral stem used in patient over 70 years. Our stem survival was comparable with survival rates reported by Zimmerer et al. and Ahmad et al. for Corail and Polarstem, respectively [33,34].

Our findings also confirmed the excellent short- to midterm outcomes reported in another previous study regarding the same prosthetic femoral implant used in younger patients [35]. Castellini et al. reported no cases of stem aseptic loosening or periprosthetic fractures, with an excellent implant survival rate (100%) with stem revision for any reason after a mean follow-up of 4 years [35]. In comparison with the study conducted by Castellini et al. our study included a larger cohort of implants from three large-volume centers, longer follow-up, a higher mean patient age, and a study population at a greater risk for perioperative complications, in order to investigate the safety and effectiveness of the same plasma-sprayed femoral stem in worse conditions with more serious indications.

Radiographically, all assessed hips appeared to have excellent osseointegration of the femoral stem. Slight signs of stress shielding were recognizable in less than 10% of the hips.

The most common intraoperative complication when using cementless stem fixation in elective THA in elderly patients, in particular female patients, or in HH for femoral neck fracture, is the fracture of the femur during press-fit implantation [36].

In the literature, the prevalence of intraoperative femoral fracture in elderly patients associated with the Corail stem ranges from 1.9% [37] to 10% [38,39]. A recent epidemiologic study on periprosthetic fracture reported 3.0% of intraoperative femoral fractures for cementless stem in primary THA [36]. In our study, although including HHs, we found a lower prevalence (0.9%) of intraoperative femoral fracture than values reported in the literature.

Postoperative periprosthetic femoral fracture, infection, and dislocation were found to be the most common postoperative complications and the main reasons for reoperation in our study. Our prevalence of postoperative periprosthetic femoral fracture was 3.2% over 10 years, confirming the fact that this remains one of the most common complications associated with cementless stems in aged patients [13].

The typical migration pattern of cementless HA-coated stems has been described as an early slight subsidence occurring in the first months after implantation followed by stem stabilization. This migration pattern does usually not imply pain or discomfort for patients, and only in rare cases does it lead to periprosthetic fracture [40].

The mean subsidence of the Corail stem has been measured to be 0.7 mm and occurred within the first 6 months, after which the implant stabilized over years [41]. Faisal et al. reported a prevalence of Corail stem subsidence greater than 2 mm of 6.3% in patients over 70 years old after THA [40]. A more recent study reported a subsidence rate of the Corail collarless stem of 3.6%, which was significantly correlated with Dorr type C femur in over 70 patients with displaced femoral neck fracture [42]. Conversely, another contemporary study reported for collared Corail stem a subsidence prevalence of 3.9% without a significant relationship with femur Dorr type [43].

The radiographic findings of the present study revealed a few cases (1.6%) of stem subsidence ≥2 mm with no associated postoperative periprosthetic fracture. This lower percentage could be explained by several factors that may play a role in reducing stem subsidence, such as the high number of Dorr type B femurs in our study population, the higher prosthetic surface roughness and friction due to the plasma-sprayed Ti coating, and accurate preoperative planning.

### Strengths and Weaknesses

The clinical relevance of this study is undoubtedly the large cohort of patients over 70 years of age receiving the same femoral stem in a multicenter clinical practice setting. Further strengths of this study are the high mean study follow-up in consideration of patient age and the high number of subjects remaining at risk at 10-year follow-up. Thus, the findings of our study could provide further evidence regarding the safety and effectiveness of the use of cementless plasma-sprayed stems in THA in elderly patients.

The current study was also not without limitations. First, the retrospective, non-controlled nature of the study design itself, which did not allow for a high level of evidence (level of evidence: 4), can be viewed as a limitation. Second, the heterogeneity of the study population and surgical treatment, which included both THA and HH, may have introduced bias into the survival analysis or complications rate. Third, only one clinical scoring system was used to assess the patient postoperative condition without a preoperative baseline. Another important limitation is the lack of a radiographic quantitative method to precisely measure with accuracy some important radiographic parameters, such as stem subsidence.

## 5. Conclusions

Our study reported 98.5% survival at 10-year follow-up for a cementless plasma-sprayed porous Ti/HA femoral stem. The lack of stem failures due to aseptic loosening, the low degree of stress shielding with excellent osseointegration, and the low incidence of intraoperative femoral fracture support the use of cementless stems in elderly patients despite postoperative femoral fracture being confirmed as one of the most common complications. On the basis of the excellent results of this study, poor bone quality and advanced age cannot be considered as contraindications for the use of this stem.

In conclusion, the use of a cementless plasma-sprayed porous Ti/HA femoral stem was proven to be a reliable and versatile option in elective primary THA for elderly patients and in HH for femoral neck fracture.

## Data Availability

Data available on request and not publicly available due to restrictions of privacy. The data presented in this study are available on request from the corresponding author and with permission of “Azienda Ospedaliera Universitaria Pisana”.
